# Physician Survey to Determine How Dengue Is Diagnosed, Treated and Reported in Puerto Rico

**DOI:** 10.1371/journal.pntd.0003192

**Published:** 2014-10-09

**Authors:** Kay M. Tomashek, Brad J. Biggerstaff, Mary M. Ramos, Carmen L. Pérez-Guerra, Enid J. Garcia Rivera, Wellington Sun

**Affiliations:** 1 Dengue Branch, Division of Vector-Borne Diseases, National Center for Emerging and Zoonotic Infectious Diseases, Centers for Disease Control and Prevention, San Juan, Puerto Rico; 2 Division of Vector-Borne Diseases, National Center for Emerging and Zoonotic Infectious Diseases, Centers for Disease Control and Prevention, Fort Collins, Colorado, United States of America; 3 Puerto Rico Department of Health, San Juan, Puerto Rico; Pediatric Dengue Vaccine Initiative, United States of America

## Abstract

Dengue is a major cause of morbidity in Puerto Rico and is well-known to its physicians. Early case identification and timely initiation of treatment for patients with severe dengue can reduce medical complications and mortality. To determine clinical management and reporting practices, and assess knowledge of dengue and its management, a survey was sent to 2,512 physicians with a medical license in Puerto Rico. Of the 2,313 physicians who received the survey, 817 (35%) completed the questionnaire. Of the respondents, 708 were currently practicing medicine; 138 were board certified (Group 1), 282 were board eligible (Group 2), and 288 had not finished residency (Group 3). Although respondents clinically diagnosed, on average, 12 cases of dengue in the preceding three months, 31% did not report any suspected cases to public health officials while about half (56%) reported all cases. Overall, 29% of respondents correctly identified early signs of shock and 48% identified severe abdominal pain and persistent vomiting as warning signs for severe dengue with the proportion of correct respondents highest in Group 1. Reportedly about sixty percent (57%) appropriately never give corticosteroids or prophylactic platelet transfusions to dengue patients. One third (30%) of respondents correctly identified administration of intravenous colloid solution as the best treatment option for dengue patients with refractory shock and elevated hematocrit after an initial trial of intravenous crystalloids, and nearly one half (46%) correctly identified administration of a blood transfusion as the best option for dengue patients with refractory shock and decreased hematocrit after a trial of intravenous crystalloids. Even though dengue has been endemic in Puerto Rico for nearly 4 decades, knowledge of dengue management is still limited, compliance with WHO treatment guidelines is suboptimal, and underreporting is significant. These findings were used to design a post graduate training course to improve the clinical management of dengue.

## Author Summary

Dengue is a major cause of morbidity in Puerto Rico and is well-known to its physicians. Early case identification and timely initiation of treatment for patients with severe dengue can reduce medical complications and mortality. We conducted a survey among physicians who practice in Puerto Rico to determine clinical management and reporting practices and assess knowledge of dengue and its management. We found that although respondents clinically diagnosed, on average, 12 cases of dengue in the preceding three months, one third did not report any suspected cases to public health officials while about half reported all cases. We found that knowledge of dengue management was limited and compliance with WHO treatment guidelines was not optimal. As other dengue endemic countries have reported similar findings, a sustained continuing medical education training initiative may be necessary to improve case detection and clinical management even in countries where the disease is common. Our findings were used to design a postgraduate training course to improve the clinical management of dengue.

## Introduction

Dengue is a mosquito-borne disease caused by any one of four dengue virus (DENV) types -1, -2, -3, and -4. Each DENV is capable of causing the full spectrum of disease from an asymptomatic infection to severe, life-threatening illness including dengue hemorrhagic fever (DHF) and dengue shock syndrome (DSS) [1]. Dengue is a major public health problem throughout the tropics and subtropics worldwide. There is currently no vaccine available to prevent dengue and vector control measures to prevent DENV transmission have not been sustainable or effective [2], [3]. Once a person has dengue, there is no licensed antiviral medication to treat or prevent severe manifestations of the disease. However, implementation of other secondary prevention measures including timely identification of dengue cases and initiation of intensive supportive treatment can reduce case fatality rates from 10% to less than 1% among severe cases [4]–[8].

The incidence and severity of dengue has been steadily increasing over the last three decades throughout much of South and Central America, Mexico, and the Caribbean including Puerto Rico [9]. Even though dengue has been endemic in Puerto Rico since the late 1960s [10], how physicians identify, diagnose, and report patients with suspected dengue is not well known. Similarly, even though the severity of dengue has increased with every subsequent dengue outbreak in Puerto Rico since 1994 [11], little is known about clinical management practices for dengue on the island. However evidence from fatal dengue case review suggests that treatment practices in Puerto Rico may differ from the World Health Organization (WHO) guidelines [12], [13] even though efforts to educate physicians concerning dengue management based on the most current guidelines had been performed from the late 1980s to the early 1990s and then intermittently with each subsequent outbreak [14]. To better understand diagnostic, treatment and reporting practices, we conducted a survey among physicians practicing in Puerto Rico in 2007–2008. Findings were used to develop a post graduate training course on the clinical management of dengue to minimize dengue morbidity and mortality, and to improve reporting of suspected dengue cases in Puerto Rico so that we can better understand the true burden of disease.

## Materials and Methods

**Figure 1:**
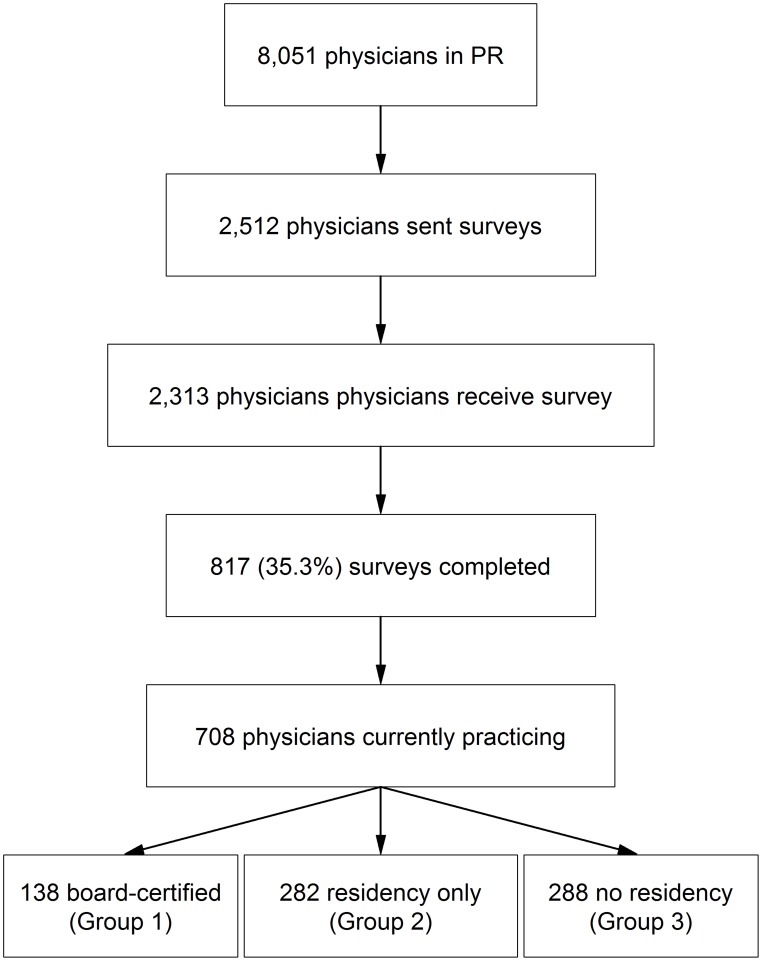
Study population. The number of physicians residing in Puerto Rico who had an active license to practice medicine is shown in the first box. A random sample of 2,512 physicians who were likely to diagnose and treat dengue patients were sent a survey as shown in the second box. Of the 2,313 physicians who received the survey (third box), 817 completed the questionnaire (fourth box). Of the 817 respondents, 109 were excluded because they were no longer practicing medicine (fifth box). For the analysis, the 708 physicians were separated into three mutually exclusive groups: board certified, residency training only, and no residency training.

In 2007, there were 8,051 physicians residing in Puerto Rico who had a Drug Enforcement Administration (DEA) number and active license to practice medicine in Puerto Rico, according to the Puerto Rico Department of Health (PRDH) (Figure 1). Physicians who were unlikely to diagnose and treat patients with dengue were excluded from the list of 8,051 physicians, including surgeons, pathologists, radiologists, allergists, dermatologists, endocrinologists, geneticists, nephrologists, neurologists, ophthalmologists, otolaryngologists, oncologists, psychiatrists, rheumatologists, and sports medicine physicians. The remaining 5,997 physicians consisted of 5,635 generalists involved in primary and emergency care of dengue patients (e.g., general practitioners, family practitioners, pediatricians, emergency department physicians, obstetricians and gynecologists, and internal medicine physicians) and 362 specialists, most notably cardiologist and pulmonologists who are most likely to work in intensive care units in Puerto Rico, and intensive care physicians.

To determine physicians' knowledge of how to diagnose and treat dengue according to the 1997 WHO guidelines and to assess their treatment and reporting practices, we determined that 1,068 generalists were needed to be 95% confident of being within ±3% of the assumed population proportion of 50%, used because this value gives the most conservative sample size estimates. We expected, based on the literature, that there would be a 50% non-participation rate, and therefore a simple random sample of 2,150 generalists was selected from the 5635 primary care and emergency medicine physicians. In addition, all 362 specialists were invited to participate. This survey underwent institutional review at the Centers for Disease Control and Prevention (CDC) and was determined to be public health practice and not research; as such, Institutional Review Board approval was not required.

We sent a 37-item questionnaire, a personalized cover letter explaining the purpose of the survey, and a pre-addressed, prepaid return envelope to the 2,512 physicians in October of 2007 (Figure 1). One hundred and ninety-nine questionnaires were returned because of an inaccurate mailing address. Three weeks after the initial mailing, a reminder postcard was sent. A second copy of the questionnaire and a pre-addressed, prepaid return envelope was sent in mid-January 2008, and a second reminder postcard was sent in early February 2008. No incentives were given for participating.

The questionnaire, which was pilot tested among a small, diverse group of physicians practicing in Puerto Rico, asked for demographic information including age, sex, and training history (e.g., location of medical school and year of graduation, location of residency training, and whether or not they were board certified or finished a residency program). Further, physicians were asked whether or not they were currently practicing medicine, the location and type of healthcare facility of their current practice, and the average number of suspected dengue patients seen per week. Respondents who reported that they were no longer practicing medicine or did not see patients with dengue were excluded from the final analysis. Respondents who were currently practicing were asked questions about the clinical and laboratory diagnosis of dengue, and how frequently they report suspected dengue cases. Practicing respondents were also asked about their hospital referral criteria for patients suspected of having dengue, and to identify warning signs for severe dengue and early signs of shock. The questionnaire also asked about specific treatment practices including use of corticosteroids, prophylactic platelet transfusions, and intravenous immune globulin for patients suspected of having dengue, and knowledge of when to use intravenous colloid solutions and blood products in dengue patients with refractory shock after an initial trial of an intravenous crystalloid. A copy of the questionnaire is available upon request.

A pre-specified analysis of management practices and knowledge of dengue was conducted by respondent level of training; groups included board-certified physicians (Group 1), residency trained physicians without board-certification (Group 2), and physicians who did not complete residency training and were therefore not qualified to take the Board examination (Group 3). Throughout, point estimates, 95% confidence intervals (CI), and statistical tests were computed accounting for the sampling design (stratified, simple random sample) and incorporating a finite population correction [15]. Comparisons among groups were made using a chi-squared test for categorical data accounting for the survey design and using the Rao-Scott adjustment [16]. Simultaneous CIs for the differences among group proportions were adjusted using the Bonferroni adjustment. Group means were compared using the likelihood ratio test with the Rao-Scott adjustment and accounting for the sampling design [16].

In order to assess the internal and external validity of the survey's results, we evaluated the questionnaires for completeness and recorded those questionnaires that were discarded and the reasons for doing so. Response proportions were compared to available population proportions for sex and physician location (San Juan metro area or Ponce), and these were assessed using a chi-squared goodness-of-fit test accounting for the sampling design [16]; other demographic information was unavailable on a population level to provide reference for evaluation. All data analyses were conducted using STATA version 10 and the survey package in R [15], [17].

In the tables and text we report both the raw counts of the numbers of respondents in the stated categories and the relevant total numbers of respondents. We also report estimates of population proportions and means based on survey data analysis, a statistical method which incorporates weighting, so these estimates may not match crude proportions calculated from the values reported. All proportions and means are such survey-based population estimates. To ease presentation, we do not include CIs in the text below when these can be found in the tables.

## Results

Of the total 2,313 physicians who received the survey, 817 (35.3%) completed the questionnaire (Figure 1). Of the 817 respondents, 109 were excluded from the final analysis because they reported that they were no longer practicing medicine. The remaining 708 physicians were separated into three mutually exclusive groups: Group 1, board certified (n = 138); Group 2, residency only (n = 282); and Group 3, no residency (n = 288) (Table 1). The majority of respondents were male, more than fifty years old, and reported attending medical school outside of Puerto Rico, mostly notably in the Dominican Republic, Spain, or Mexico. Respondents reported practice locations throughout the island. The proportion of male respondents (62%) differed significantly, if not dramatically, from the reported physician population proportion of males (69%) (p = 0.01), while the rates for physician office location did not (San Juan metro area, p = 0.42; Ponce, p = 0.48).

**Table 1:** Physician characteristics overall and by level of training group.

	All responders (N = 817)Group 1 (N = 138)Group 2 (N = 282)Group 3 (N = 288)	(p-value)
Characteristic	No.: Estimated Percentage* (95% CI)				
**Male gender**	500: 64(61,67)	81: 58(50,66)	173: 64(58,69)	178: 65(60,71)	0.42
**Age, years**					
21–40	93: 11(9,14)	21: 15(10,22)	32: 11(8,15)	40: 14(11,18)	
41–50	220: 27(24,30)	51: 41(33,50)	94: 34(29,39)	65: 23(19,28)	
51–60	277: 34(31,38)	49: 37(29,45)	101: 36(31,41)	108: 38(33,43)	
61+	217: 27(24,30)	15: 8(5,11)	53: 19(15,24)	71: 25(21,30)	<0.001
**Year Graduated**					
1990s+	154: 20(17,23)	29: 21(15,29)	56: 21(17,26)	64: 23(19,28)	
1980s	305: 40(37,43)	63: 53(44,61)	115: 43(38,49)	113: 41(36,47)	
1970s	214: 27(24,30)	36: 22(17,30)	70: 26(21,31)	77: 28(23,33)	
Prior to 1970	103: 13(11,15)	7: 4(2,6)	27: 10(7,14)	19: 7(5,10)	0.14
**Medical School**					
Puerto Rico	271: 30(27,33)	107: 81(75,86)	101: 36(31,41)	30: 10(7,14)	
Dominican Republic	216: 29(27,32)	7: 4(2,9)	71: 25(21,30)	125: 43(38,49)	
Spain	166: 22(19,24)	6: 3(2,6)	59: 21(17,21)	63: 22(18,27)	
Mexico	110: 14(12,17)	7: 5(2,10)	40: 14(11,18)	58: 20(16,25)	
USA	19: 2(1,3)	5: 3(1,5)	3: 1(0.4,3)	3: 1(0.4,3)	
Other	35: 4(3,6)	6: 4(2,8)	8: 3(1,5)	9: 3(2,6)	<0.001
**Practice Site**					
San Juan Metro	310: 43(39,46)	80: 58(49,66)	131: 47(41,52)	99: 35(30,40)	
Ponce Area	42: 6(5,8)	8: 7(4,14)	23: 8(6,12)	11: 4(2,7)	
Other	346: 51(47,54)	48: 35(27,43)	126: 45(39,50)	172: 61(56,66)	<0.001

* Percentages incorporate survey design weights, and thus may not equal the crude proportions. Fewer than 12% of respondents failed to answer any individual question; the denominator includes only those who answered the question in order to give the most conservative estimate.

Respondent characteristics varied by group (Table 1). Groups 1 and 2 had roughly similar age distributions, but a higher proportion of Group 1 respondents attended medical school in Puerto Rico and reportedly practiced medicine in the San Juan Metro Area when compared to Group 2 or 3 respondents. Group 3 respondents were more likely than those from Group 1 and 2 to be older, trained in Dominican Republic, and practicing outside of the San Juan Metro Area.

Respondents clinically diagnosed on average 12 cases of dengue in the three months before participating in the survey (Table 2). Slightly more than half (56%) of all respondents stated that they report all clinically suspected dengue cases to public health officials while about one third (31%) said that they do not report any suspected cases. During this same time period, respondents requested dengue diagnostic testing for only three cases on average. Few respondents were able to correctly identify laboratory assays used to diagnose acute DENV infections, however, this varied significantly by group with Group 1 respondents being most likely to respond correctly (Table 3).

**Table 2:** Clinical and laboratory diagnosis and reporting of dengue patients.

	Overall (n = 708)Group 1 (n = 138)Group 2 (n = 282)Group 3 (n = 288)	Overall p-value
Number of diagnoses in last 3 months	Estimated Mean (95% CI)				
Clinical diagnoses	11.5(10.3,12,7)	10.9(8.1,13.7)	11.5(9.7,13.3)	11.6(9.8,13.5)	0.93
Laboratory diagnoses	3.0(2.4,3.6)	2.2(1.4,2.9)	2.5(1.7,3.2)	4.0(2.8,5.2)	0.02

Number of suspected cases reported	Number: Estimated Proportion (95% CI)				Overall p-value
All cases	368: 56(52,50)	62: 46(37,54)	156: 60(54,65)	150: 55(50,61)	
Some cases	85: 13(10,15)	21: 20(13,28)	28: 11(7,15)	36: 13(10,17)	
No cases	207: 31(28,35)	44: 35(27,44)	77: 30(25,35)	86: 32(27,37)	0.13

**Table 3:** Knowledge of early signs of shock and warning signs for severe dengue, how to make laboratory diagnosis and reported criteria for referral to hospital.*

	Overall (n = 708)Group 1 (n = 138)Group 2 (n = 282)Group 3 (n = 288)	p-value
	No./Total No. Who Answered: Estimated Percentage (95% CI)				
**Clinical diagnosis (% who always use):**					
Use 1997 WHO case definition†	624/678: 92(90,94)	15/128: 88(80,93)	255/273: 93(90,96)	254/277: 92(88,94)	0.20
Use platelet count	654/685: 96(94,97)	122/130: 95(90,97)	265/278: 95(92,97)	267/277: 96(94,98)	0.66
Use white cell count	600/680: 89(86,91)	111/130: 86(80,91)	245/275: 89(85,92)	244/275: 89(84,92)	0.76
Use tourniquet test	119/626: 19(16,22)	19/126: 11(8,16)	48/253: 19(5,24)	52/247: 21(17,26)	0.07
**Knowledge (% who correctly identified):**					
Tachycardia & delayed capillary refill as early sign of shock	215/708: 29(26,33)	54/138: 38(30,46)	101/282: 36(31,41)	60/288: 21(17,26)	<0.001
Severe abdominal pain and persistent vomiting as warning signs	347/708: 48(44,51)	86/138: 63(55,71)	132/282: 47(41,52)	129/288: 45(39,50)	0.003
All warning signs listed†	181/665: 26(23,30)	44/126: 35(27,44)	75/266: 28(23,33)	62/274: 23(18,28)	0.04
All laboratory tests used to diagnose acute cases of dengue	40/578: 6(4,7)	21/119: 15(10,22)	16/232: 7(4,11)	3/227: 1(0.5,4)	<0.001
**Hospital Referral Criteria (% who refer for):**					
Criteria consistent with 1997 Guidelines	216/677: 31(28,34)	47/125: 33(25,41)	101/275: 37(31,42)	68/277: 25(20,30)	0.002
Minor bleeding without shock or hemoconcentration	457/677: 68(64,71)	74/125: 58(49,67)	189/275: 69(63,74)	194/277: 70(65,75)	0.02
Platelet count ≤100,000 without bleeding, hemoconcentration, or shock	213/677: 32(28,35)	32/125: 24(17,32)	106/275: 38(33,44)	75/277: 27(22,32)	<0.001

* Percentages incorporate survey design weights, and thus may not equal the crude proportions. Fewer than 12% of respondents failed to answer any individual question; the denominator includes only those who answered the question in order to give the most conservative estimate.

† 1997 WHO case definition defined dengue as an acute febrile illness of 2 to 7 days duration with 2 or more of the following: headache, retro-orbital pain, myalgia, arthralgia, rash, hemorrhagic manifestations, leucopenia. Warning signs include: severe abdominal pain, persistent vomiting, cold and clammy skin/extremities, narrowing pulse pressure, hypotension, change in mental status (e.g., irritability, lethargy).

Methods used to clinically diagnose patients with dengue did not differ by group (Table 3). The majority (92%) reported always using criteria consistent with the 1997 WHO case definition to identify suspected dengue cases while a similar proportion (96%) reportedly always use platelet count or white cell count (89%) to identify suspected cases. Less than one quarter (19%) of all respondents reported using the tourniquet test to identify suspected dengue cases.

Knowledge of warning signs for severe dengue and early signs of shock was low overall and knowledge varied by group (Table 3). One-third (29%) of respondents overall correctly identified tachycardia and delayed capillary refill as early signs of shock, and this proportion increased from Group 3 to Group 1. One half (48%) of all respondents were able to correctly identify severe abdominal pain and persistent vomiting as warning signs with a higher proportion of Group 1 respondents than Group 2 or 3 respondents being able to do so. Ability to identify all warning signs from a list was low (26%), with Group 1's ability significantly higher than Groups 2 and 3 (p = 0.04).

About one third (31%) of respondents reportedly use hospital referral criteria consistent with the 1997 guidelines (Table 3). Not all respondents reportedly refer suspected dengue patients who have a hemorrhagic manifestation; 68% of all respondents refer suspected dengue patient with minor bleeding (e.g., epistaxis, gum bleeding) in the absence of shock or hemoconcentration. About one third (32%) of all respondents use a platelet count of ≤100,000 cells/mm^3^ in the absence of bleeding, hemoconcentration, or shock as a criteria for hospital referral.

Knowledge of the WHO treatment guidelines varied among groups (Table 4). When given the scenario of a suspected dengue patient with persistent shock and an elevated hematocrit level after a trial of an intravenous crystalloid solution, one third (30%) of all respondents correctly responded that they would give the patient an intravenous colloid. This proportion varied from 39 to 23% among groups with Group I having the highest proportion of correct answers. However, a higher proportion of Groups 1 (48%) and 2 (47%) respondents said that they would give the patient a vasopressor given this scenario. Given a second scenario where a suspected dengue patient has persistent shock and a decreasing hematocrit level after a trial of intravenous crystalloids, about half (46%) of all respondents correctly identified blood transfusion as the treatment of choice, and the proportions among the groups were not statistically significantly different.

**Table 4:** Reported knowledge of and adherence to 1997 World Health Organization treatment guidelines.*

	Overall (n = 708)Group 1 (n = 138)Group 2 (n = 282)Group 3 (n = 288)	p-value
	No./Total No. Who Answered: Estimated Percentage (95% CI)				
**Knowledge (% who would first give):**					
Intravenous colloid solution for refractory shock with elevated hematocrit	100/307: 30(26,35)	36/82: 39(29,50)	46/145: 32(25,39)	18/80: 23(15,32)	0.07
Vasopressors for above scenario	129/307: 42(37,48)	36/82: 48(37,59)	68/145: 47(38,54)	25/80: 31(23,41)	0.02
Blood transfusion for refractory shock with decreased hematocrit	143/300: 46(40,51)	46/79: 53(42,64)	63/139: 46(38,53)	34/82: 41(32,52)	0.33
Vasopressors for last scenario	79/300: 27(22,32)	17/79: 22(14,33)	45/139: 33(26,40)	17/82: 21(14,30)	0.06
**Actual Practice (% who never give):**					
Corticosteroid	389/666: 57(53,61)	92/127: 72(64,80)	148/266: 56(50,61)	149/273: 55(49,60)	0.006
Prophylactic platelet transfusion	90/152: 57(49,64)	34/48: 70(56,81)	43/83: 51(41,61)	13/21: 62(42,79)	0.14
Intravenous immunoglobulin	322/351: 92(89,94)	80/84: 97(93,99)	134/156: 86(80,91)	108/111: 97(92,99)	<0.001

*Percentages incorporate survey design weights, and thus may not equal the crude proportions.

Many (57%) of the respondents appropriately never give corticosteroids to their patients with suspected dengue (Table 4). The majority (72%) of Group I respondents reportedly do not use corticosteroids while slightly more than half of Group 2 and 3 respondents reported not giving corticosteroids to their suspected dengue patients. Likewise, the same proportion (57%) of respondents correctly does not give prophylactic platelet transfusions, and this practice varied in a similar fashion by group. Among the 63 respondents who reportedly give prophylactic platelet transfusions, 41 (65%) individuals stated that their threshold for giving platelets is between 25,000 and 50,000 cells/mm^3^, while 22 (35%) gave ≤20,000 cells/mm^3^ as their threshold. The overwhelming majority (92%) of respondents appropriately do not give intravenous immunoglobulin to their patients with dengue.

## Discussion

This survey demonstrates that knowledge and management of dengue vary among physicians practicing in Puerto Rico, particularly between Board-certified physicians and non-Board-certified physicians, especially those who did not complete residency training. There were four important findings from this survey. First, while most reportedly use WHO case definition to clinically diagnose dengue cases, we found that case reporting to public health authorities is not optimal and knowledge of laboratory diagnosis of dengue was poor. Second, many respondents, regardless of their level of training, were unable to identify early signs of shock and warning signs for severe dengue, knowledge needed to effectively give anticipatory guidance and inform triage and referral decisions. Third, reported compliance with treatment guidelines of dengue patients in refractory shock was generally low. Fourth, corticosteroids and prophylactic platelet transfusions were reportedly used by about 40% of respondents; practices that are not recommended by current or past treatment guidelines [13], [18]–[20].

While respondents reportedly had clinically diagnosed 12 dengue cases on average in the preceding three months, about one-third of respondents did not report any cases to the Puerto Rico Department of Health and half reported all cases as required by law. Taken together, these findings suggest that dengue is underreported in Puerto Rico. This finding is consistent with past studies that estimated that for every case of suspected dengue reported to the passive dengue surveillance system (PDSS) in Puerto Rico, ten to 27 cases are not reported, and for every case of dengue hemorrhagic fever (DHF) reported, 15 DHF cases are not reported [21], [22]. Given these findings, it was not surprising to find that few (∼6%) respondents knew which laboratory tests are used to diagnose acute dengue as they do not routinely report cases to PDSS, a system that requires submission of a case investigation form and serum sample for case reporting and free diagnostic testing.

Treatment guidelines for the clinical management of dengue were first introduced by WHO in 1975 [18] and they were then updated in 1997 [13] and 2009 [19]. The 1997 WHO guidelines were translated into Spanish, widely distributed throughout the Caribbean, and in use when this survey was administered (October 2007 to February 2008) [20]. Identification of dengue patients with early signs of shock and warning signs for severe dengue and timely initiation of supportive care is the cornerstone of dengue clinical management. Survey respondents, regardless of their level of training, were largely unable to identify early signs of shock and warning signs for severe dengue. Moreover, because of their lack of knowledge, most respondent's reported hospital referral criteria deviated from WHO guidelines. Those guidelines recommended that dengue patients without bleeding or warning signs could be monitored at home by family members while clinicians monitor platelet count and hematocrit as an outpatient. Suspected dengue patients with any hemorrhagic manifestation and patients with a platelet count <100,000 cells per mm^3^ concurrent with an elevated hematocrit for age and sex were to be referred to a hospital for further evaluation. Dengue patients with signs of shock and/or warning signs were to be referred for inpatient hospitalization. Consistent with these findings fatal case review studies conducted in Puerto Rico have found missed opportunities for referral and hospital admission [12], [23].

Both the 1997 guidelines and the current 2009 WHO guidelines have comparable treatment algorithms for the use of intravenous crystalloids, colloids, and blood transfusions in dengue patients with refractory shock. Findings from our survey suggest that these treatment algorithms, especially the use of intravenous colloids for refractory shock due to severe plasma leakage, may not be as widely used as should be. In the same year as the survey was conducted, a medical record review from a case-series of laboratory-positive fatal dengue cases in Puerto Rico found that only one patient was given an intravenous colloid solution before the terminal event even though six of eight case-patients who died in the hospital had refractory shock [12]. This is noteworthy because application of the WHO treatment guidelines have been associated with a reduction in case fatality rates from 10 to less than 1% among patients with severe dengue [4]–[8].

Even though WHO guidelines [13], [18]–[20] and a 2006 Cochrane review [24] recommend against the use of corticosteroids in patients with dengue, 43% of respondents reported prescribing corticosteroids, a finding corroborated by the 2007 fatal dengue case-series that found that 55% of fatal laboratory-positive dengue cases were given a corticosteroid [12]. This practice also occurs in other dengue endemic countries [25], [26]. Reasons given by respondents for use of corticosteroids included use as an immune modulator given that severe manifestations of dengue are thought, in part, to be immune mediated (CDC, data not presented). However, a recent randomized clinical trial evaluating the early use of oral prednisolone in dengue patients found treatment to have little impact on the host immune response [27]. In addition, while the trial was not powered to assess efficacy, there was no evidence that treatment lead to a reduction in the severity of plasma leakage, or the development of shock or clinical bleeding. In short, with no evidence of therapeutic benefit and multiple potential side effects including hyperglycemia, immunosuppression, secondary infections, and gastrointestinal bleeding in critically ill patients, corticosteroids should not be used to treat patients with dengue [24], [27].

Despite a lack of evidence, many of our survey respondents reported giving prophylactic platelet transfusions to their patients with dengue; a practice that may be relatively common among physicians in dengue endemic countries [25], [28]. Several studies have found no correlation between platelet count and bleeding or bleeding severity in patients with dengue, and when given, prophylactic platelet transfusions do not expedite platelet recovery [29]–[32]. Moreover, the practice is costly and may contribute to fluid overload and the development of pulmonary edema resulting in increased hospital stays among dengue patients. A recent randomized control trial suggested that prophylactic platelet transfusions have no therapeutic benefit when given to patients with dengue and they may be associated with adverse outcomes including transfusion reactions [33]. A clinical trial is currently ongoing to further evaluate the use of prophylactic platelet transfusions among patients with dengue (ClinicalTrials.gov identifier NCT01030211).

Although these findings contribute to our understanding of the knowledge and management of dengue in Puerto Rico, our study has several limitations. First, non-response might have biased the results [34], [35]. Demographic and training characteristics of respondents and non-respondents were similar in most respects, though there was somewhat higher response by females, but we do not expect that this or other differences likely relate to their level of knowledge and practice. Second, our survey relied on self-reported practices and the accuracy of this information is not known. Previous studies suggest that physicians often over state their compliance with clinical guidelines when compared with chart review [36], [37]. An evaluation is ongoing to confirm actual practice patterns for hospitalized dengue patients in Puerto Rico. Lastly, differences in knowledge and practices we found among physician groups may be explained by non-Board certified physicians having less contact with severe dengue patients. However, there was no difference among the groups in the average number of clinical diagnoses made in the three months before participating in the survey.

In summary, our survey suggests that despite dengue being endemic in Puerto Rico for more than 40 years, physicians' diagnosis and clinical management of dengue in Puerto Rico are not optimal. As other dengue endemic countries have reported similar findings, a sustained continuing medical education training initiative may be necessary to improve case detection and clinical management even in countries where the disease is common [25], [28]. Findings from this survey were used to develop and implement a post graduate clinical management course attended by more than 8,000 physicians licensed to practice in Puerto Rico in 2010 and create an on-line version of the course that was released in March of 2014. Further study is needed to determine if focused training can improve clinical management by minimizing failed early recognition of severe dengue and delayed initiation of supportive care that can result in higher rates of medical complications, longer hospital stays, and increased hospital costs. An evaluation of the course and its impact on the clinical management of dengue in Puerto Rico is ongoing.
